# The Inclusion of Whole-Fat Dairy in the 2025–2030 Dietary Guidelines for Americans: Nutritional Evidence, Health Outcomes, and Policy-Industry Implications

**DOI:** 10.3390/foods15213926

**Published:** 2026-11-02

**Authors:** Xindi Guo, Ziqi Li, Tianyi Sun, Guohui Shen, Tiannuo Hong, Zisen Chen, Mingxue Yu, Jindong Zhang, Miao Wang, Fengyue Xu, Qining Liu, Yanting Gu, Lu Chen, Yanzhi Guo

**Affiliations:** 1 Institute of Food and Nutrition Development, Ministry of Agriculture and Rural Affairs, Beijing 100081, China; c1ndyg@163.com (X.G.); usk1029@163.com (Z.L.); shenguohuicaas@163.com (G.S.); htn163ah@163.com (T.H.); an1399120911@163.com (Z.C.); mingxue.yu@doct.uliege.be (M.Y.); kingdong924@163.com (J.Z.); 15022075207@163.com (M.W.); 18903800783@163.com (F.X.); 821012551048@caas.cn (Q.L.); guyanting2022@163.com (Y.G.); 2 Faculty of Arts and Education, The University of Auckland, Auckland 1142 HB, New Zealand; suntianyi291@126.com; 3 Environmental Economics and Natural Resources Group, Wageningen University and Research, 6700 Wageningen, The Netherlands; 4 Gembloux Agro-Bio Tech, University of Liège, 5030 Gembloux, Belgium; 5 Chinese Academy of Agricultural Sciences, Beijing 100081, China

**Keywords:** Dietary Guidelines for Americans, whole-fat dairy, food matrix, health outcomes, policy

## Abstract

The 2025–2030 Dietary Guidelines (DGA) for Americans explicitly include whole-fat dairy products without added sugars, challenging the long-standing preference for low-fat and fat-free dairy. This review examines the scientific basis and broader implications of this change by integrating evidence on nutritional composition, dairy food structure, and human health outcomes. Reducing dairy fat generally lowers total fat, saturated fat, and energy density, but its effects on micronutrient composition, milk-fat-associated components, and food structure are more complex and vary by product type and processing. Human evidence has likewise not consistently associated whole-fat dairy with poorer cardiovascular, metabolic, growth-related, musculoskeletal, or gastrointestinal outcomes, although current evidence does not establish a general health advantage over reduced-fat alternatives. Together, these findings suggest that dairy-fat content alone provides an incomplete basis for evaluating dairy foods. Product type, food matrix, processing, dietary substitution, and population context should also be considered when interpreting dairy-related health effects and formulating dietary recommendations. The updated DGA is therefore better understood as an expansion of acceptable dairy choices rather than a reversal of existing saturated-fat guidance, with important implications for dairy product development and nutrition communication.

## 1. Introduction

Dairy products are important contributors to dietary nutrient intake, providing high-quality protein, calcium, phosphorus, potassium, iodine, and several essential nutrients [1,2]. They are therefore widely incorporated into dietary guidelines as part of healthy eating patterns. Recommendations concerning dairy consumption, however, have long distinguished products according to milk-fat content, particularly in relation to saturated fat and energy intake [3,4,5,6,7,8,9,10,11,12].

The historical preference for low-fat and fat-free dairy products developed within dietary strategies aimed at reducing total fat and saturated fat intake [13]. Because milk fat contains a substantial proportion of saturated fatty acids, reduced-fat dairy products offered a practical means of maintaining dairy-derived protein, minerals, and other nutrients while lowering saturated fat and energy consumption [14,15]. This rationale shaped dairy recommendations for several decades and remained evident in the 2020–2025 Dietary Guidelines for Americans (DGA), which continued to emphasize fat-free and low-fat milk, yogurt, and cheese [11,16].

The 2025–2030 DGA introduced an important change by explicitly including whole-fat milk, yogurt, and cheese without added sugars among acceptable dairy options. At the same time, the guideline retained the recommendation that saturated fat contribute less than 10% of total energy intake [12]. The updated guidance therefore broadens the range of dairy-fat choices without removing saturated fat as a relevant nutritional consideration.

This change coincides with an expanding body of evidence showing that dairy foods differ in ways that are not fully captured by fat content alone [17,18]. Reducing dairy fat generally lowers saturated fat and energy density, but other nutritional characteristics vary according to product type, fortification, processing, fermentation, and food structure. Milk, yogurt, cheese, butter, and other dairy products also differ substantially in their physical matrices and in the organization of proteins, lipids, minerals, and bioactive components [17,19]. These distinctions may contribute to differences in digestion, metabolism, and health outcomes among dairy products with comparable or differing fat contents [2,20]. Human evidence has likewise complicated a simple low-fat versus whole-fat comparison [21,22]. Lower dairy-fat intake may improve selected intermediate risk factors, particularly saturated fat exposure and some blood lipid measures, yet whole-fat dairy has not been consistently associated with poorer cardiovascular, metabolic, growth-related, or gastrointestinal outcomes [23]. These findings do not establish a general health advantage of whole-fat dairy, but they raise questions about whether dairy foods can be adequately evaluated or ranked according to fat content alone.

In this review, we evaluate the scientific basis and implications of the changes in dairy recommendations in the 2025–2030 DGA. We first summarize the historical evolution of dairy-fat recommendations and then evaluate how fat reduction affects the nutritional composition and structural characteristics of dairy foods. Further, we assess evidence linking dairy-fat level and specific dairy products with cardiovascular, metabolic, growth-related, musculoskeletal, and gastrointestinal outcomes. Finally, we consider the scientific interpretation and boundaries of the policy change, its implications for dairy product development and nutrition communication, and the major evidence gaps that should guide future research.

## 2. Evolution of Dairy Recommendations in the Dietary Guidelines for Americans

The Dietary Guidelines for Americans (DGA) represent one of the most influential frameworks shaping dietary recommendations and nutrition policies in the United States and have influenced other food-based dietary guidelines (FBDG) worldwide. Dairy foods have consistently been recognized as an important component of healthy dietary patterns because they provide high-quality protein, calcium, phosphorus, potassium, iodine, and several vitamins. However, recommendations regarding dairy fat have changed considerably across successive editions of the DGA [3,4,5,6,7,8,9,10,11,12].

As summarized in Table 1, one of the most notable changes over the historical development of the DGA has been the evolution of recommendations concerning dairy-fat content. Earlier editions progressively established a preference for fat-free and low-fat dairy products, reflecting concerns about total fat, saturated fat, and energy intake. In the 2025–2030 DGA, this long-standing emphasis was broadened by explicitly including whole-fat milk, yogurt, and cheese without added sugars among acceptable dairy choices. Thus, the major change is not simply the introduction of whole-fat dairy, but a broader range of dairy-fat levels that can be accommodated within a healthy dietary pattern.

At the same time, the definition of the Dairy Group has also evolved, including changes in the treatment of lactose-free products and fortified soy beverages and soy yogurt as nutritionally comparable alternatives. Other aspects of dairy guidance, including recommended amounts, added sugars, and specific nutrient considerations, have also changed across editions. These developments provide important context for understanding the evolution of dairy recommendations, while the shift in dairy-fat guidance represents the most substantive change in how the nutritional characteristics of dairy products have been distinguished across recent editions.

### 2.1. Historical Rationale for the Preference for Low-Fat and Fat-Free Dairy Products

The long-standing preference for low-fat and fat-free dairy products developed within broader dietary strategies aimed at reducing total fat and saturated fat intake [24]. During the late twentieth century, nutrition recommendations were strongly influenced by a nutrient-reduction framework that emphasized limiting dietary components considered to contribute to chronic disease risk [25]. Saturated fatty acids and dietary cholesterol were therefore major targets in strategies to improve cardiovascular health [26].

Because milk fat contains a relatively high proportion of saturated fatty acids, whole-fat dairy products were historically considered potential contributors to unfavorable lipid profiles, particularly through increases in low-density lipoprotein cholesterol (LDL-C) [26]. Reducing dairy fat consequently became a practical strategy for lowering saturated fat exposure.

Low-fat and fat-free dairy products were considered appropriate alternatives because they retained many of the nutritional benefits of dairy foods while providing less total fat, saturated fat, and energy [11]. Removal of milk fat substantially reduces lipid-derived energy, whereas many non-fat milk components, including high-quality proteins, calcium, phosphorus, lactose, potassium, and other nutrients, remain largely preserved [17].

Within the prevailing scientific framework of the time, recommendations favoring low-fat or fat-free dairy products therefore offered a practical way to maintain dairy consumption while supporting population-level goals for reducing saturated fat intake.

### 2.2. Dairy Recommendations in the 2020–2025 Dietary Guidelines for Americans

The 2020–2025 DGA maintained the long-standing preference for fat-free and low-fat dairy products. Within the Healthy U.S.-Style Dietary Pattern, the Dairy Group included fat-free or low-fat milk, yogurt, and cheese, as well as lactose-free versions of these products and fortified soy beverages and soy yogurt as nutritionally comparable alternatives.

This emphasis on reduced-fat dairy was consistent with the broader recommendation to limit saturated fat intake to less than 10% of total daily energy intake. The 2020–2025 DGA encouraged replacing foods higher in saturated fat with foods containing unsaturated fats or lower-fat alternatives, making dairy-fat reduction one practical approach for meeting this goal.

Low-fat and fat-free dairy products thus offered a practical means of maintaining dairy-related nutrient intake while reducing saturated fat exposure. This preference also extended beyond individual dietary advice. School meal programs and other public nutrition initiatives incorporated reduced-fat dairy products, contributing to their widespread availability in institutional settings [27,28].

Although the range of dairy-related options had broadened through the inclusion of lactose-free products and fortified soy alternatives, recommendations regarding milk-fat content remained centered on fat-free and low-fat dairy foods.

### 2.3. Changes in the 2025–2030 Dietary Guidelines: Inclusion of Whole-Fat Dairy Products

The 2025–2030 DGA introduced a notable change by explicitly including whole-fat dairy products without added sugars as acceptable components of healthy dietary patterns. In contrast to previous editions that primarily emphasized fat-free and low-fat dairy products, the updated guidelines include fat-free, low-fat, and whole-fat milk, yogurt, and cheese. This marks a substantive change from the previous emphasis on milk-fat level as a major basis for distinguishing recommended dairy choices.

The updated recommendation does not remove existing guidance on saturated fat. The 2025–2030 DGA continues to recommend limiting saturated fat consumption to less than 10% of total energy intake. The inclusion of whole-fat dairy products should therefore be interpreted within the context of overall dietary patterns rather than as a general recommendation to increase dairy fat or saturated fat intake.

The qualifier “without added sugars” further indicates that dairy foods are not evaluated solely according to their naturally occurring fat content. Product formulation and other compositional characteristics remain relevant to overall dietary quality.

The recommended amount of dairy also remains approximately three servings per day within a 2000 kcal dietary pattern [12]. The principal change is therefore an expansion of acceptable dairy choices, rather than an increase in recommended dairy intake or a replacement of low-fat and fat-free products.

### 2.4. Literature Search and Evidence Selection

Literature searches were conducted in PubMed in July 2026 using combinations of dairy-related terms (dairy, milk, yogurt/yoghurt, cheese, and kefir), fat-content terms (whole-fat, full-fat, regular-fat, low-fat, reduced-fat, skim/skimmed, fat-free, and non-fat), and outcome-specific keywords. These keywords covered cardiovascular health (CVD, CHD, stroke, blood lipids, and blood pressure), obesity and metabolic health (obesity, BMI, metabolic syndrome, type 2 diabetes, and insulin resistance), growth and musculoskeletal health (bone mineral density, fracture, osteoporosis, growth, muscle mass, and sarcopenia), and gastrointestinal health (gut microbiota, SCFA, bowel function, intestinal barrier, and inflammation). Search terms were also combined with terms related to observational studies, clinical trials, and evidence syntheses.

The four searches retrieved a total of 3541 records, including 1002 cardiovascular, 1218 metabolic, 1029 musculoskeletal, and 292 gastrointestinal records. Titles and abstracts were screened, followed by full-text assessment of potentially relevant publications. Non-human studies, non-research publications, and studies that lacked relevant dairy exposures or health outcomes, or did not provide separately extractable dairy-related results, were excluded. The final evidence sets comprised 90 cardiovascular, 28 musculoskeletal, 9 gastrointestinal, and 6 metabolic publications.

## 3. Beyond Fat Content: Nutritional and Structural Heterogeneity of Dairy Products

Milk-fat content has traditionally provided a practical basis for distinguishing whole-fat, low-fat, and non-fat dairy products, and this classification captures an important nutritional difference: reducing milk fat predictably lowers total fat, saturated fatty acids (SFA), and energy density. However, fat reduction does not alter all components of dairy foods to the same extent. Protein, minerals, water-soluble vitamins, fat-soluble nutrients, milk-fat-associated bioactive components, and processing-induced food structures respond differently to fat removal and subsequent processing. Moreover, these effects vary substantially among fluid milk, yogurt, cheese, and other dairy products. Therefore, understanding the nutritional implications of dairy-fat reduction requires consideration of both fat level and the broader compositional and structural characteristics of specific dairy foods.

To provide illustrative comparisons, 14 dairy food records were selected from a manually compiled set of 73 records in USDA FoodData Central (SR Legacy and Foundation Foods). Nutrient data for these 14 records were obtained from SR Legacy (April 2018 release; accessed on 30 April 2026) and presented individually per 100 g edible portion (Table 2 and Table 3). Original food descriptions and selection details are provided in Appendix A Table A1 and Table A2. These U.S. food composition records are intended for illustrative comparison and may not represent the range of dairy products available in other countries.

### 3.1. Macronutrient Changes Associated with Dairy-Fat Reduction

The most direct consequence of dairy-fat reduction is a decrease in total fat and SFA, generally accompanied by lower energy density. This relationship is particularly clear in fluid milk, where products with different fat levels are otherwise relatively comparable in composition. In the USDA data, whole milk contained 3.27 g fat, 1.86 g SFA, and 61 kcal per 100 g, compared with 0.18 g total fat, 0.12 g SFA, and 35 kcal in nonfat milk, while low-fat milk showed intermediate values (Table 2). In contrast, protein, carbohydrate, and total sugar contents were broadly similar across these categories (Table 2). Thus, fat removal from fluid milk primarily reduces lipid-derived energy and SFA exposure while largely preserving the major non-fat macronutrients.

This compositional effect provides a clear nutritional basis for the historical preference for low-fat and fat-free milk when reducing saturated fat and energy intake has been a primary dietary objective. Importantly, however, the relationship between fat level and macronutrient composition becomes less straightforward in other dairy categories. Yogurt composition may vary because of fermentation, concentration, protein fortification, and formulation, whereas cheese composition is strongly affected by coagulation, whey removal, moisture loss, salting and ripening [2,20]. Because the data are expressed per 100 g, differences in moisture and total solids may also influence apparent nutrient concentrations, particularly in cheese (Table 2). Consequently, differences observed between low-fat and whole-fat products may reflect not only fat removal but also differences in product formulation and processing.

### 3.2. Micronutrient Changes Following Fat Removal and Processing

Unlike total fat, SFA, and energy, most micronutrients do not follow a consistent gradient across dairy-fat categories. Minerals such as calcium, phosphorus, and potassium, together with water-soluble vitamins like riboflavin and vitamin B_12_ are primarily associated with the aqueous phase and non-fat milk solids and are therefore largely retained after fat removal [17,20]. In the selected USDA fluid-milk records, differences in these nutrients among whole, low-fat, and non-fat products were substantially smaller than the corresponding differences in fat and energy (Table 3).

Variation becomes greater when different dairy categories are considered. Fermentation, concentration, whey separation, salting, and ripening can alter micronutrient retention and concentration independently of fat content. Cheese, for example, may contain high concentrations of casein-associated calcium and phosphorus because of curd formation and moisture loss, while potassium and some water-soluble components may be lost with whey. Thus, micronutrient density reflects product type and processing characteristics in addition to milk-fat level [29,30].

Fat-soluble vitamins require a different interpretation. Vitamins A and E and carotenoids are naturally associated with the lipid fraction [31,32], and their intrinsic concentrations may decrease with milk-fat removal. However, the vitamin content of commercial dairy products is also strongly influenced by fortification practices, animal feeding, production conditions, processing, and analytical methods. In the United States, reduced-fat and nonfat milk may be fortified with vitamins A and D; consequently, higher measured concentrations of these vitamins in some low-fat or nonfat products should not be interpreted as a direct consequence of fat removal. Conversely, whole-fat products should not be assumed to contain uniformly higher concentrations of all vitamins and minerals. The micronutrient comparison therefore leads to a different conclusion from the macronutrient comparison. Low-fat and nonfat dairy products generally retain many of the micronutrients associated with non-fat milk solids, whereas differences in fat-soluble vitamins are strongly modified by fortification and processing. Milk-fat level alone is consequently an incomplete indicator of the micronutrient quality of dairy foods.

### 3.3. Milk-Fat-Associated Bioactive Components Affected by Fat Removal

Evaluating dairy fat solely in terms of total fat and SFA also overlooks the compositional complexity of the milk-fat fraction. In natural milk, triglycerides are organized within milk fat globules (MFGs) surrounded by the milk fat globule membrane (MFGM), a structurally complex interface containing phospholipids, sphingolipids, cholesterol, membrane proteins, and other bioactive constituents [33]. Milk fat also contains characteristic fatty acids, including short- and medium-chain fatty acids, branched- and odd-chain fatty acids, conjugated linoleic acid, and small amounts of n-3 polyunsaturated fatty acids [14,34,35].

Reducing milk fat lowers triglycerides and SFA and can alter the amounts and distribution of other milk-fat-associated constituents [33,36]. This distinction is important because whole-fat dairy products are not simply reduced-fat products with additional saturated fat; they also differ in the amounts and organization of lipid-associated constituents. At the same time, the relationship is not strictly proportional to total fat content. Separation, homogenization, thermal treatment, and fermentation may disrupt the native MFGM, alter milk-fat-globule size, and redistribute membrane-associated components between lipid and aqueous phases. Therefore, a higher total fat content does not necessarily indicate greater preservation of the native milk-fat structure [33,36]. These observations do not establish that whole-fat dairy products are nutritionally superior to reduced-fat products. Rather, they indicate that reducing dairy fat changes more than SFA exposure alone. The amount, composition, and physical organization of milk-fat-associated components must also be considered when interpreting differences between dairy products with different fat levels.

### 3.4. Processing-Related Changes in Dairy Food Matrix Structure

Milk-fat level represents only one dimension of dairy-food composition. Processing transforms the organization of proteins, lipids, minerals, water, and microbial components and thereby creates distinct dairy food matrices. Fluid milk is an oil-in-water emulsion in which milk fat globules coexist with casein micelles and soluble nutrients. During yogurt fermentation, acidification reorganizes casein micelles into a protein gel that entraps fat globules, water, and microorganisms. Cheese production involves coagulation and whey removal, producing a denser protein-mineral matrix in which fat is embedded within the curd. In contrast, churning and phase inversion generate the fat-continuous structure of butter, which contains considerably less protein and mineral material than milk, yogurt, or cheese [2,15,20].

These structural differences are nutritionally relevant because they determine how dairy components are spatially organized and released during digestion [2,20]. Consequently, the physiological effects of a given amount of dairy fat may vary depending on whether that fat is consumed within milk, yogurt, cheese, butter, or another dairy matrix. Evidence from controlled feeding studies illustrates this principle: dairy fat incorporated within an intact cheese matrix has produced different lipid responses from comparable amounts of dairy fat provided as butter or as separately assembled dairy components [37]. Such findings do not identify a single “beneficial” matrix component, but they demonstrate that the metabolic meaning of dairy fat cannot necessarily be inferred from its fatty-acid content alone.

Fermentation adds another layer of heterogeneity. Yogurt, kefir, and other fermented dairy products contain fermentation-derived metabolites and, depending on the product, viable microorganisms [38,39]. Their nutritional and physiological characteristics therefore differ from those of fluid milk even when total fat contents are comparable. Similarly, butter should not be treated as nutritionally equivalent to whole milk or cheese simply because all contain milk-derived saturated fat.

Dairy-fat classification captures nutritionally relevant differences in total fat and SFA content, and often energy density, but it does not fully characterize the nutritional composition or structural organization of dairy foods. Fat reduction may leave many non-fat nutrients largely intact, alter the amounts of fat-associated constituents, and interact with processing in ways that differ among dairy categories. Accordingly, whole-fat, low-fat, and nonfat products should not be interpreted as homogeneous groups independent of product type. This compositional and structural heterogeneity provides an essential basis for interpreting the health outcome evidence discussed in the following section.

## 4. Dairy Fat Content and Human Health Outcomes

Low-fat and fat-free dairy products were historically prioritized in part because reducing dairy fat generally lowers SFA content and energy density. A central question in evaluating dairy-fat recommendations is whether these compositional differences consistently translate into more favorable health outcomes. Our assessment of the current evidence, as presented in the following subsections, is that it does not show that whole-fat dairy products are uniformly associated with a greater risk of adverse outcomes across cardiovascular, metabolic, musculoskeletal, or gastrointestinal domains. At the same time, the available evidence does not establish a general health advantage of whole-fat over reduced-fat dairy. The effects observed in human studies vary according to dairy category, dietary context, and the health outcome considered.

### 4.1. Dairy Fat and Cardiovascular Health

Reducing dairy fat intake can lower SFA intake and may improve selected blood lipid biomarkers. In a 24-week randomized trial involving children, replacing regular-fat dairy with reduced-fat alternatives was associated with a 0.15 mmol/L lower LDL-C concentration than in the control group at week 24 [21]. An earlier short-term study also reported reductions in total cholesterol and LDL-C during skim-milk consumption [40]. Direct comparisons between products with different fat levels, however, have produced less consistent results. Whole milk did not adversely affect total cholesterol, LDL-C, or triglycerides relative to skim milk, but increased HDL-C in a short-term trial [41]. Similarly, regular-fat cheese did not produce a less favorable LDL-C response than reduced-fat cheese [42]. The form in which dairy fat is consumed may also matter: dairy fat incorporated within an intact whole-fat cheese matrix resulted in lower total and LDL cholesterol than did comparable nutrients provided as reduced-fat cheese plus butter or as separately assembled components [37].

Evidence for clinical cardiovascular outcomes is similarly more complex than would be predicted from SFA content alone. Meta-analyses have reported inverse associations of low-fat dairy with hypertension (RR = 0.94, 95% CI: 0.90–0.98, per 200 g/day) [22] and stroke (RR = 0.91, 95% CI: 0.85–0.97, highest versus lowest intake) [43], whereas findings for high-fat dairy have been less consistent across studies [44,45]. These differences have not consistently extended to coronary heart disease, overall cardiovascular disease, or mortality. Systematic reviews and large prospective cohorts generally report neutral or modestly favorable associations for dairy intake across several product categories, including milk, yogurt, and cheese [46,47]. A recent Norwegian cohort study also reported an association between higher whole-milk intake and a higher risk of ischemic heart disease mortality (HR = 1.13, 95% CI: 1.01–1.27, compared with no intake) [48]. Studies specifically examining higher dairy-fat products have likewise not shown a consistent increase in cardiovascular risk [46,47]. Some studies have associated high-fat milk intake with higher cardiovascular or mortality risk, but these findings have not been consistent across products or populations [49].

Reduced-fat dairy may therefore remain useful when lowering SFA intake or LDL-C is a specific nutritional objective. However, current evidence does not show that whole-fat dairy products are consistently associated with higher cardiovascular risk. Concentrated dairy fats such as butter should also be considered separately from dairy foods in which fat is embedded within protein- and mineral-rich matrices, as controlled comparisons have reported higher LDL-C concentrations following butter consumption than following cheese consumption despite similar SFA intake [50].

### 4.2. Dairy Fat and Weight-Related Metabolic Health

The higher energy density of whole-fat dairy has raised concerns regarding body weight and obesity, yet observational and intervention studies in adults have not consistently shown greater adiposity among consumers of whole-fat dairy. Prospective studies and meta-analyses generally report neutral or inverse associations between whole-fat dairy intake and changes in body weight, waist circumference, or obesity risk, with yogurt showing one of the more consistent favorable associations [22,51]. Similar findings have been observed in children. Whole-fat dairy intake has not been consistently associated with greater adiposity, and the Milky Way trial found no significant difference in body fat or body composition between whole-fat and reduced-fat dairy interventions [23,52].

Additional energy from whole-fat dairy may promote weight gain when added without compensatory changes elsewhere in the diet. In an eight-week randomized crossover trial, supplementing habitual diets with whole-fat dairy resulted in 1.2 kg greater weight gain than supplementing with low-fat dairy [53]. Evidence for type 2 diabetes mellitus (T2DM) and metabolic syndrome (MetS) also does not support a simple adverse effect of whole-fat dairy. Earlier studies showed inverse associations with low-fat dairy [54,55], whereas the PURE study reported lower MetS prevalence with higher whole-fat dairy intake (OR = 0.72, 95% CI: 0.66–0.78, at least two servings/day versus none) [56]. Yogurt has shown more consistent inverse associations with T2DM and MetS in observational studies, although evidence that these associations differ by fat level remains limited [22,57].

Current evidence does not consistently indicate that whole-fat dairy intake is associated with an increased risk of obesity, T2DM, or MetS. Energy balance, product type, added sugars, and the broader dietary pattern should also be considered when evaluating associations between dairy-fat level and metabolic outcomes.

### 4.3. Dairy Fat in Growth and Musculoskeletal Development

Evidence directly comparing low-fat and whole-fat dairy products for growth, bone health, and musculoskeletal outcomes remains limited. In children, dairy consumption may support nutrient adequacy, growth, and lean-mass development [58,59], but available observational and randomized studies have not demonstrated consistent differences in body composition according to fat level [23,60].

Milk and fortified reduced-fat dairy products can also contribute to bone mineral accrual and maintenance through their protein, calcium, and, when fortified, vitamin D [61,62,63]. Studies of dairy-related muscle outcomes have largely focused on protein intake and resistance exercise [64,65]. An umbrella review reported generally favorable associations between dairy consumption and bone mineral density, but inconsistent findings for fracture risk [66]. Although inflammation can affect bone remodeling and dietary saturated fat has been implicated in inflammatory pathways [67], clinical evidence has not established that whole-fat dairy increases bone resorption through this mechanism [68]. Because direct comparisons of nutritionally matched dairy products differing mainly in fat content remain limited, current evidence is insufficient to establish milk-fat level as a major determinant of growth or musculoskeletal outcomes. Selection of dairy-fat level in these contexts may therefore depend more on energy requirements, nutritional status, and life stage.

### 4.4. Dairy Fat and Gastrointestinal Health

For gastrointestinal outcomes, dairy product characteristics other than fat content appear to play a particularly important role. Yogurt and kefir can improve lactose digestion and gastrointestinal tolerance relative to milk, with these effects largely attributable to fermentation and microbial lactase activity rather than dairy-fat level [69]. Studies of low-fat yogurt and whole-fat dairy products have reported variable effects on gut microbial composition, but these studies did not directly compare fat levels and therefore cannot establish whether dairy-fat content independently affects overall microbial diversity or fecal short-chain fatty acid concentrations [70,71,72]. Experimental evidence from mice indicates that high-fat diets can alter gut microbial composition and predicted metabolic pathways, although these findings cannot be directly extrapolated to whole-fat dairy consumption in humans [73]. Evidence concerning intestinal barrier function and inflammation remains limited and heterogeneous. Studies of low-fat yogurt, whole-fat yogurt, and MFGM-enriched dairy products have noted variable effects on endotoxin exposure, intestinal permeability, inflammation markers, and microbial metabolites [72,74,75]. Because these interventions also differ in fermentation, food matrix, and comparator foods, available studies do not establish a consistent gastrointestinal advantage of either low- or whole-fat dairy products.

Across these outcome domains, reducing dairy fat generally lowers SFA content and often energy density, but these compositional differences do not consistently translate into superior health outcomes. Cardiovascular evidence shows the clearest effects of fat reduction on selected intermediate risk factors, whereas differences in obesity risk and metabolic, musculoskeletal, and gastrointestinal outcomes are less consistently related to fat level. The effects of dairy consumption also vary among milk, yogurt, cheese, butter, and fermented products, indicating that dairy-fat content should be interpreted together with product type and dietary context when evaluating health outcomes. These findings provide the evidentiary context for interpreting why the updated DGA broadened the range of acceptable dairy-fat choices while retaining existing limits on saturated fat intake.

## 5. Scientific Interpretation of the Updated Dairy Recommendation

The evidence reviewed in the preceding sections does not support a simple hierarchy in which lower dairy-fat content necessarily indicates greater nutritional value or more favorable health effects. At the same time, it does not justify replacing reduced-fat dairy with whole-fat products as a general dietary strategy. The updated recommendation is therefore best interpreted in light of these two observations: dairy fat remains nutritionally relevant, but fat content alone is insufficient to determine the role of a dairy food within a healthy dietary pattern.

### 5.1. Evidence Supporting a Broader Range of Dairy-Fat Choices

Reducing dairy fat predictably lowers saturated fat and energy intake, but these compositional differences do not consistently translate into superior health outcomes across all dairy products and health domains. This distinction is central to interpreting the 2025–2030 DGA: the evidence challenges the assumption that lower dairy-fat content necessarily indicates greater overall healthfulness, but it does not establish that whole-fat dairy is universally superior to reduced-fat alternatives. The updated recommendation can therefore be understood as allowing greater flexibility in dairy selection while retaining the need to consider product composition, nutritional requirements, and the overall dietary pattern. The rationale for broadening dairy-fat choices is thus not to replace reduced-fat products, but to avoid treating fat content as the sole criterion for evaluating dairy foods.

### 5.2. Limits of the Updated Recommendation

The inclusion of whole-fat dairy should not be interpreted as a relaxation of all concerns surrounding saturated fat. The 2025–2030 DGA continues to recommend limiting saturated fat intake to less than 10% of total energy, and the amount contributed by whole-fat dairy remains relevant when assessing the overall dietary pattern including SFA from other food sources.

Nor does the updated recommendation reduce the value of low-fat and fat-free dairy products. These foods continue to provide high-quality protein, calcium, phosphorus, and other essential nutrients with lower energy density and saturated fat content. They may remain particularly useful when energy restriction or saturated fat reduction is a priority.

The recommendation also needs to be interpreted at the level of specific foods. Whole milk, plain whole-fat yogurt, and cheese should not be grouped indiscriminately with butter, cream-rich foods, or sugar-sweetened dairy products simply because they all contain dairy fat. Their nutrient density, food matrix, degree of concentration, and dietary roles differ substantially. The specification of “without added sugars” reinforces the importance of considering the overall formulation of the product rather than focusing on fat content in isolation.

The appropriate choice of dairy-fat level may also vary among individuals and populations. Age, energy requirements, habitual dietary intake, nutritional status, and cardiometabolic risk can alter the relative importance of energy density and saturated fat exposure. Population-level flexibility therefore does not imply that all dairy-fat choices are nutritionally equivalent in every context.

### 5.3. Toward a Broader Framework for Evaluating Dairy Foods

The changes in dairy recommendations are also relevant to a broader question in nutrition science: how should complex foods be evaluated when the effects of individual nutrients do not fully predict the effects of the foods that contain them?

Nutrient-based evaluation remains essential. Saturated fat, added sugars, energy density, protein, vitamins, and minerals all provide important information about the nutritional characteristics of dairy products. The limitation arises when one of these attributes is used as the dominant basis for ranking foods whose compositions and physical structures differ substantially.

Dairy foods illustrate this problem particularly well. Their nutritional properties arise from combinations of nutrients and structural features that vary with processing and product type. Fermentation reorganizes the milk matrix and introduces microbial and fermentation-derived components; cheese production concentrates proteins and minerals while embedding fat within a dense casein matrix; and butter represents a much more concentrated fat source with a markedly different composition. These distinctions cannot be captured adequately by milk-fat percentage alone.

A broader evaluation of dairy foods therefore does not replace nutrient-based guidance. Rather, it places individual nutrient characteristics within the context of the whole food and the dietary pattern in which that food is consumed. The updated inclusion of whole-fat dairy is consistent with this approach: saturated fat remains an important consideration, but it is no longer the sole characteristic used to define whether a dairy food can fit within a healthy dietary pattern.

## 6. Implications for the Dairy Industry

The broader inclusion of whole-fat dairy products in the 2025–2030 DGA may have implications for dairy product development and nutrition communication. The significance for the dairy industry lies not in replacing low-fat products with whole-fat alternatives, but rather in expanding the range of dairy products that can be positioned within healthy dietary patterns. This may create greater scope for product differentiation based on nutritional composition, processing characteristics, and intended dietary use.

### 6.1. Implications for Dairy Product Development

For the U.S. dairy industry, the updated recommendation may shift the focus of product evaluation beyond fat content alone. Whole milk, whole-fat yogurt, and traditional cheese can be considered alongside low-fat and fat-free products rather than being evaluated primarily according to their saturated fat content.

This broader perspective may encourage product development that emphasizes multiple nutritional characteristics rather than fat reduction alone. Products may be differentiated according to attributes such as the absence of added sugars, high-quality protein, fermentation, nutrient density, and product structure. Fermented dairy products, high-protein products, and products with relatively simple formulations may therefore receive greater attention as part of a more diversified dairy portfolio.

The same principle may also increase flexibility in institutional settings such as schools and public nutrition programs, where dairy choices have historically been strongly influenced by reduced-fat recommendations. Any expansion of available options, however, will continue to depend on overall nutrient composition, energy contribution, and the nutritional needs of the target population.

### 6.2. Implications for the Chinese Dairy Industry

The policy evolution in the United States may offer broader lessons for dairy industries internationally, particularly regarding product development and consumer communication. Although the U.S. policy debate highlights how dairy products should be evaluated beyond fat content alone, the implications for other countries depend on their dietary recommendations and consumption patterns. China is a particularly relevant case because it presents a complementary challenge: while the United States has debated the role of dairy fat in dietary guidance, China continues to face substantial gaps between recommended and actual dairy consumption. This contrast allows the review to examine how evolving dairy recommendations may inform both product differentiation and efforts to improve overall dairy intake in different nutritional contexts.

China’s Dietary Guidelines for Chinese Residents (2022) recommend consuming 300–500 g of milk and dairy products daily, an increase from the 300 g recommended in the 2016 guidelines [76,77]. However, actual consumption remains substantially below this target. In 2021, per capita dairy consumption in China was 42.3 kg annually, approximately one-third of the global average [78]. A systematic review found that milk and yogurt were the most commonly consumed dairy products among Chinese adults, with intake varying by age, sex, region, and urban-rural differences and generally higher in urban, northern, eastern, and coastal areas [79]. Evidence indicates that dairy intake remains inadequate among Chinese children aged 3–8 years, with only about one-third consuming dairy products and nearly all falling below recommended intake, alongside nutrient gaps in calcium and potassium, with consumption varying by household income, maternal education, and urban-rural residence [80]. These findings suggest that the Chinese dairy industry needs to address low intake and unequal access alongside product differentiation, rather than focusing on fat content alone.

For Chinese dairy enterprises, the greater value of this policy development may lie in supporting a more differentiated approach to product design. Low-fat and fat-free products may remain appropriate for consumers seeking lower energy or saturated fat intake, while whole-fat dairy products may be suitable for other nutritional needs, sensory preferences, or consumption contexts. Product development could therefore emphasize a combination of nutritional characteristics, including added sugar content, protein content, and fermentation, while improving familiar products such as milk and yogurt. Given the predominance of milk and yogurt in current consumption, improving the nutritional quality and affordability of these familiar products may be particularly relevant, while expanding product variety could provide additional options for consumers with different needs [79]. Consumer communication should likewise explain the nutritional characteristics of different products without presenting fat content as the sole indicator of healthfulness. The appropriate balance among these product categories should, however, be guided by evidence generated within the Chinese dietary and population context.

### 6.3. Socioeconomic and Environmental Considerations

The implications of the evolving U.S. dietary recommendations depend not only on nutritional considerations but also on socioeconomic and cultural factors that shape access to and acceptance of different dairy products, as well as the environmental consequences of changes in dairy consumption.

Access and affordability may vary across communities: a nationwide study of U.S. food stores found that fewer than half stocked 1% and skim milk, compared with more than three-quarters stocking whole and 2% milk. The availability of low-fat milk was also lower in low-income and some minority communities [81]. Consumer preferences are equally relevant. In a study conducted in four supermarkets in Philadelphia, 40% of participants reported typically purchasing whole milk and 38% purchased 2% milk [82]. Although these findings are specific to the settings studied and do not establish current nationwide price differences, they illustrate how local availability, relative prices, and established preferences may influence responses to revised dietary guidance. Consequently, broadening recommendations to include whole-fat dairy does not necessarily mean that consumers will adopt these products uniformly, as dietary choices remain shaped by household budgets, retail access, and familiarity with different milk types.

These issues are particularly important in school meal programs and food assistance initiatives, where decisions about milk provision must balance budget constraints with the needs and preferences of participants. Although the inclusion of whole-fat milk may be economically feasible, differences in local supply and students’ taste preferences may affect its practical implementation and acceptance.

Environmental sustainability is another important consideration in evaluating the shift toward whole-fat dairy in the U.S. dietary recommendations. Existing life-cycle assessment data indicate that whole and skim milk have similar greenhouse gas emissions per kilogram of product, whereas skim milk has higher emissions per unit of energy. Conversely, whole milk has slightly higher emissions per gram of protein [83]. These differences highlight the complexity of assessing the environmental implications of shifting toward whole-fat dairy, as the relative impacts vary across nutritional and environmental considerations. The overall environmental impact of the revised recommendations will also depend on consumer responses. Substitution between whole-fat and reduced-fat milk is not necessarily one-to-one, as purchasing decisions are influenced by relative prices and consumer preferences [84], while differences in product availability may further affect consumption patterns. Therefore, their sustainability implications should be assessed alongside nutritional and health outcomes, taking into account both environmental impacts and real-world consumption patterns.

### 6.4. Nutrition Communication and Future Product Positioning

The updated recommendation also raises an important issue for health communication. Continuing to characterize whole-fat dairy as inherently unfavorable would oversimplify the available evidence, whereas presenting whole-fat dairy as generally healthier than reduced-fat alternatives would be equally unsupported. Communication should instead reflect differences among specific dairy products and place fat content within the context of overall nutritional quality.

This distinction is particularly important because whole milk, plain whole-fat yogurt, cheese, butter, cream-based products, and sugar-sweetened dairy foods differ substantially in nutrient density, food matrix, and dietary role. Their nutritional value cannot be inferred from dairy-fat content alone, and policy changes concerning whole-fat dairy should not be extended indiscriminately to all foods containing dairy fat.

Future product positioning should therefore be guided more strongly by evidence directly related to product composition, nutritional function, and intended use. Greater transparency in product formulation and closer alignment between health claims and nutritional evidence may help reduce oversimplification in both product development and consumer communication.

For both the U.S. and Chinese dairy industries, the central implication is diversification rather than substitution. Future dairy development may increasingly depend on matching different product characteristics with specific nutritional needs and dietary contexts, rather than defining product value according to a single attribute such as fat content.

## 7. Limitations of Current Evidence and Future Research Directions

Despite the broader acceptance of whole-fat dairy products in the 2025–2030 DGA, important uncertainties remain regarding the optimal role of dairy fat within healthy dietary patterns. Current evidence challenges a simple ranking of dairy products according to fat content, but it is not yet sufficient to define a universally preferable dairy-fat level across products, populations, and dietary contexts.

### 7.1. Limitations of Current Evidence

A major limitation is the predominance of observational evidence. Prospective cohort studies have provided important information on associations between dairy consumption and long-term health outcomes, but residual confounding, dietary measurement error, and differences in lifestyle or overall dietary patterns remain difficult to exclude. Randomized trials are generally shorter in duration and more often evaluate intermediate biomarkers than clinical disease outcomes.

Comparisons between whole-fat and reduced-fat dairy products are also complicated by differences in formulation. Fat reduction may be accompanied by changes in added sugars, protein concentration, stabilizers, fortification, or other ingredients, meaning that two products differing in labeled fat content may not otherwise be nutritionally equivalent. More tightly controlled comparisons are needed to isolate the effects of dairy-fat level from those of product reformulation.

An equally important issue is the choice of comparator or replacement food. The health effects associated with reducing whole-fat dairy may differ substantially depending on whether it is replaced by low-fat dairy, refined carbohydrates, or foods rich in unsaturated fat. Failure to specify these substitutions can contribute to apparently conflicting conclusions across studies.

Finally, dairy production systems, fortification practices, habitual dairy intake, and overall dietary patterns differ among countries and populations. However, the present review draws predominantly on evidence from Western populations, with limited representation of studies from Asia, Africa, and Latin America. This geographic imbalance reflects the scope of the available evidence considered in the review and limits the extent to which the findings can be generalized to populations with different dietary and socioeconomic contexts. China is specifically highlighted in the future research agenda because of its large population, distinct dietary patterns, and dairy-consumption practices that differ from those in Western countries, making it an important context for evaluating the applicability of current evidence. Evidence generated largely from Western cohorts may therefore have limited generalizability to populations with different dairy-consumption patterns, nutritional status, or metabolic characteristics.

### 7.2. Future Research Priorities

Future research should place greater emphasis on direct, product-specific comparisons. Trials comparing whole-fat and reduced-fat versions of the same dairy product, with comparable protein, carbohydrate, micronutrient, and ingredient profiles, would help distinguish effects related specifically to dairy fat. Milk, yogurt, cheese, and other fermented products may also be evaluated separately rather than being treated as interchangeable members of a single dairy category.

Longer-term intervention studies are needed to complement observational evidence. In addition to conventional lipid and glycemic markers, future studies should investigate clinically relevant outcomes and intermediate pathways that may help explain product-specific responses. Explicitly defined substitution designs will be particularly important for determining whether the effects of dairy-fat reduction depend on the foods or nutrients that replace it.

Mechanistic research should further clarify how milk-fat-associated structures, proteins, minerals, fermentation-derived components, and the food matrix interact during digestion and metabolism. Such research may help explain why dairy products with similar total fat or SFA contents can produce different physiological responses and may identify which structural characteristics are most relevant to human health.

Greater population diversity is also needed to assess the generalizability of current evidence and its implications for dietary guidance across different populations. For China, studies should consider habitual dairy consumption levels, lactose tolerance, dietary patterns, and population-specific metabolic characteristics when evaluating different dairy products. Evidence from Chinese cohorts and intervention studies will be important for determining the extent to which findings from Western populations can inform local dietary recommendations.

Future research should therefore move beyond attempts to identify a universally preferable dairy-fat level and instead determine how fat content interacts with product type, food structure, dietary substitution, and population characteristics. Such evidence will be essential for developing dietary guidance that moves beyond a single fat-based classification while remaining grounded in the nutritional characteristics of specific dairy foods and the dietary contexts in which they are consumed.

## 8. Conclusions

The current evidence supports a more differentiated interpretation of dairy fat than one based on fat content alone. Reducing dairy fat reliably lowers total fat, saturated fat, and energy intake, while largely preserving many non-fat nutrients, including protein and minerals. However, dairy products also differ in milk-fat-associated components, fermentation-derived constituents, processing characteristics, and food matrices. These differences are reflected in health literature: whole-fat dairy has not been consistently associated with poorer cardiovascular, metabolic, growth-related, musculoskeletal, or gastrointestinal outcomes, although existing evidence does not establish a general health advantage over reduced-fat alternatives.

Against this evidence base, the inclusion of whole-fat milk, yogurt, and cheese without added sugars in the 2025–2030 DGA is better understood as a broadening of acceptable dairy choices rather than a reversal of saturated-fat guidance or the establishment of a new preference for whole-fat products. The change reflects a more product-specific approach in which dairy-fat content remains relevant but is considered together with nutrient composition, food structure, processing, dietary context, and population needs. For the Chinese dairy industry, this development may provide a useful reference for product diversification, but its application should remain grounded in local dietary patterns and population-specific nutritional needs.

## Figures and Tables

**Table 1:** Evolution of Dairy Recommendations Across Editions of the Dietary Guidelines for Americans.

DGA Edition	Dairy Recommendation
1980	Consume adequate amounts of milk and dairy products while avoiding excessive fat, saturated fat, and cholesterol intake.
1985	Choose foods that provide adequate calcium and other nutrients while limiting fat and saturated fat intake.
1990	Choose a diet low in fat, saturated fat, and cholesterol; select low-fat milk and dairy products more often.
1995	Choose low-fat or fat-free milk, yogurt, and cheese as part of a healthy diet.
2000	Consume 2–3 servings from the Milk Group daily and choose low-fat or fat-free dairy products.
2005	Consume 3 cups per day of fat-free or low-fat milk or equivalent milk products.
2010	Increase intake of fat-free or low-fat milk and milk products such as milk, yogurt, and cheese.
2015–2020	Fat-free or low-fat dairy, including milk, yogurt, cheese, and/or fortified soy beverages, is recommended as part of healthy eating patterns.
2020–2025	Dairy foods include fat-free or low-fat milk, yogurt, and cheese, as well as lactose-free versions and fortified soy beverages and soy yogurt.
2025–2030	Dairy foods may include fat-free, low-fat, and whole-fat milk, yogurt, and cheese without added sugars, as well as lactose-free versions and fortified soy alternatives.

**Table 2:** Macronutrient composition of selected dairy products by milk-fat level (per 100 g edible portion).

Dairy Product	Milk-Fat Category	Energy (kcal)	Total Fat (g)	SFA (g)	Protein (g)	Carbohydrate (g)	Total Sugars (g)	Water (g)	Dry Matter (g)
Fluid milk	Nonfat	35.0	0.18	0.12	3.37	4.86	5.09	90.84	9.16
	Low-fat (1%)	42.0	0.97	0.63	3.37	4.99	5.20	89.92	10.08
	Reduced-fat (2%)	50.0	1.98	1.26	3.30	4.80	5.06	89.21	10.79
	Whole (3.25%)	61.0	3.27	1.86	3.15	4.78	5.05	88.13	11.87
Plain yogurt	Skim	56.0	0.18	0.12	5.73	7.68	7.68	85.23	14.77
	Low-fat	63.0	1.55	1.00	5.25	7.04	7.04	85.07	14.93
	Whole milk	61.0	3.25	2.10	3.47	4.66	4.66	87.90	12.10
Greek yogurt	Nonfat	59.0	0.39	0.12	10.19	3.60	3.24	85.10	14.90
	Low-fat	73.0	1.92	1.23	9.95	3.94	3.56	83.56	16.44
	Whole milk	97.0	5.00	2.40	9.00	3.98	4.00	81.30	18.70
Mozzarella, low-moisture	Part-skim	295.0	19.78	11.29	23.75	5.58	1.90	47.07	52.93
	Whole milk	318.0	24.64	15.56	21.60	2.47	1.01	48.38	51.62
Ricotta	Part-skim	138.0	7.91	4.93	11.39	5.14	0.31	74.41	25.59
	Whole milk	150.0	10.18	6.42	7.54	7.27	0.27	73.69	26.31

Note: Each row represents one USDA food record, not a mean of multiple products. SFA, saturated fatty acids; dry matter = 100 − water (g/100 g). Fluid-milk records are specified as without added vitamins A and D. Milk-fat descriptors follow USDA product names. Values are illustrative food composition data, not results from matched experimental products. Source: USDA FoodData Central, SR Legacy (April 2018 release), FDC IDs are shown for traceability.

**Table 3:** Micronutrient composition of selected dairy products by milk-fat level (per 100 g edible portion).

Dairy Product	Milk-Fat Category	Ca (mg)	P (mg)	K (mg)	Mg (mg)	Na (mg)	Zn (mg)	Vitamin A (µg RAE)	Vitamin D (µg)	Vitamin B12 (µg)	Riboflavin (mg)
Fluid milk	Nonfat	122.0	101.0	156.0	11.0	42.0	0.42	2.0	0.00	0.50	0.18
	Low-fat (1%)	125.0	95.0	150.0	11.0	44.0	0.42	14.0	0.00	0.47	0.18
	Reduced-fat (2%)	120.0	92.0	140.0	11.0	47.0	0.48	28.0	0.00	0.53	0.18
	Whole (3.25%)	113.0	84.0	132.0	10.0	43.0	0.37	46.0	0.10	0.45	0.17
Plain yogurt	Skim	199.0	157.0	255.0	19.0	77.0	0.97	2.0	0.00	0.61	0.23
	Low-fat	183.0	144.0	234.0	17.0	70.0	0.89	14.0	0.00	0.56	0.21
	Whole milk	121.0	95.0	155.0	12.0	46.0	0.59	27.0	0.10	0.37	0.14
Greek yogurt	Nonfat	110.0	135.0	141.0	11.0	36.0	0.52	1.0	0.00	0.75	0.28
	Low-fat	115.0	137.0	141.0	11.0	34.0	0.60	90.0	0.00	0.52	0.23
	Whole milk	100.0	135.0	141.0	11.0	35.0	0.52	2.0	0.00	0.75	0.28
Mozzarella, low-moisture	Part-skim	697.0	548.0	188.0	27.0	666.0	3.62	227.0	0.40	1.68	0.35
	Whole milk	575.0	412.0	75.0	21.0	710.0	2.46	197.0	0.50	0.73	0.27
Ricotta	Part-skim	272.0	183.0	125.0	15.0	99.0	1.34	107.0	0.10	0.29	0.18
	Whole milk	206.0	154.0	219.0	20.0	110.0	0.53	120.0	0.20	0.85	0.30

Note: Each row represents one USDA food record; no cross-product averages are calculated. RAE, retinol activity equivalents; vitamin D, D2 + D3. Values may reflect product formulation, analytical methods, fortification, and moisture/solids in addition to milk-fat content. Source: USDA FoodData Central, SR Legacy (April 2018 release); FDC IDs are shown for traceability.

## Data Availability

No new data were created or analyzed in this study. Data sharing is not applicable to this article.

## Abbreviations

The following abbreviations are used in this manuscript:

DGA	Dietary Guidelines for Americans
FBDG	Food-Based Dietary Guidelines
LDL-C	Low-Density Lipoprotein Cholesterol
CVD	Cardiovascular Disease
CHD	Coronary Heart Disease
BMI	Body Mass Index
SCFA	Short-Chain Fatty Acid
USDA	United States Department of Agriculture
SFA	Saturated fatty acids
MFGs	Milk fat globules
MFGM	Milk fat globule membrane
HDL-C	High-density lipoprotein cholesterol
RR	Risk Ratio
CI	Confidence Interval
HR	Hazard Ratio
T2DM	Type 2 diabetes mellitus
MetS	Metabolic syndrome
OR	Odds Ratio

## Appendix A

**Table A1:** Identification and characteristics of selected dairy food records shown in Table 2 and Table 3.

Product Subgroup	Fat Description	Original USDA Food Description	Total Fat (g/100 g)	Formulation/Description Note
Fluid milk	Nonfat	Milk, nonfat, fluid, without added vitamin A and vitamin D (fat free or skim)	0.18	Without added vitamins A and D (explicit in USDA title)
	Low-fat (1%)	Milk, fluid, 1% fat, without added vitamin A and vitamin D	0.97	Without added vitamins A and D (explicit in USDA title)
	Reduced-fat (2%)	Milk, reduced fat, fluid, 2% milkfat, without added vitamin A and vitamin D	1.98	Without added vitamins A and D (explicit in USDA title)
	Whole (3.25%)	Milk, whole, 3.25% milkfat, without added vitamin A and vitamin D	3.27	Without added vitamins A and D (explicit in USDA title)
Plain yogurt	Skim	Yogurt, plain, skim milk	0.18	Fortification/additive status not explicitly specified in USDA title
	Low-fat	Yogurt, plain, low fat	1.55	Fortification/additive status not explicitly specified in USDA title
	Whole milk	Yogurt, plain, whole milk	3.25	Fortification/additive status not explicitly specified in USDA title
Greek yogurt	Nonfat	Yogurt, Greek, plain, nonfat (Includes foods for USDA’s Food Distribution Program)	0.39	USDA Food Distribution Program mentioned; fortification not specified
	Low-fat	Yogurt, Greek, plain, lowfat	1.92	Fortification/additive status not explicitly specified in USDA title
	Whole milk	Yogurt, Greek, plain, whole milk	5.00	Fortification/additive status not explicitly specified in USDA title
Low-moisture mozzarella	Part-skim	Cheese, mozzarella, low moisture, part-skim	19.78	Low-moisture style specified; added ingredients not detailed
	Whole milk	Cheese, mozzarella, whole milk, low moisture	24.64	Low-moisture style specified; added ingredients not detailed
Ricotta	Part-skim	Cheese, ricotta, part skim milk	7.91	Fortification/additive status not explicitly specified in USDA title
	Whole milk	Cheese, ricotta, whole milk	10.18	Fortification/additive status not explicitly specified in USDA title

Note: Each row is one USDA food record, not a group mean. USDA fat descriptors are taken from the food titles; they are not based on a common threshold across products. Absence of fortification information in a title does not establish that a product is unfortified. Source: USDA FoodData Central, SR Legacy (April 2018).

**Table A2:** Traceability of compiled USDA dairy food records and illustrative display decisions.

FDC ID	Official USDA Food Description	Database	Revised Product Subgroup	Original Fat Classification	Original Analysis Scope	Presentation Rationale	Total Fat (g/100 g)	USDA Record URL
170863	Cheese, American, nonfat or fat free	SR Legacy	American (cheese)	nonfat	baseline	Not selected	0	https://fdc.nal.usda.gov/food-details/170863/nutrients (accessed on 30 April 2026)
171295	Cheese, cheddar, nonfat or fat free	SR Legacy	Cheddar (cheese)	nonfat	baseline	Not selected	0	https://fdc.nal.usda.gov/food-details/171295/nutrients (accessed on 30 April 2026)
171292	Cheese, cheddar, reduced fat (Includes foods for USDA’s Food Distribution Program)	SR Legacy	Cheddar (cheese)	reduced fat	baseline	Not selected	20.41	https://fdc.nal.usda.gov/food-details/171292/nutrients (accessed on 30 April 2026)
173417	Cheese, cottage, lowfat, 1% milkfat	SR Legacy	Cottage cheese (cheese)	lowfat/1% milkfat	baseline	Not selected	1.02	https://fdc.nal.usda.gov/food-details/173417/nutrients (accessed on 30 April 2026)
167703	Cheese, cottage, lowfat, 1% milkfat, lactose reduced	SR Legacy	Cottage cheese (cheese)	lowfat/1% milkfat	baseline	Not selected	1	https://fdc.nal.usda.gov/food-details/167703/nutrients (accessed on 30 April 2026)
168114	Cheese, cottage, lowfat, 1% milkfat, no sodium added	SR Legacy	Cottage cheese (cheese)	lowfat/1% milkfat	baseline	Not selected	1	https://fdc.nal.usda.gov/food-details/168114/nutrients (accessed on 30 April 2026)
167716	Cheese, cottage, lowfat, 1% milkfat, with vegetables	SR Legacy	Cottage cheese (cheese)	lowfat/1% milkfat	sepcific processed	Not selected	1	https://fdc.nal.usda.gov/food-details/167716/nutrients (accessed on 30 April 2026)
328841	Cheese, cottage, lowfat, 2% milkfat	Foundation	Cottage cheese (cheese)	lowfat/2% milkfat	baseline	Not selected	2.3	https://fdc.nal.usda.gov/food-details/328841/nutrients (accessed on 30 April 2026)
172182	Cheese, cottage, lowfat, 2% milkfat	SR Legacy	Cottage cheese (cheese)	lowfat/2% milkfat	baseline	Not selected	2.27	https://fdc.nal.usda.gov/food-details/172182/nutrients (accessed on 30 April 2026)
172181	Cheese, cottage, nonfat, uncreamed, dry, large or small curd	SR Legacy	Cottage cheese (cheese)	nonfat	baseline	Not selected	0.29	https://fdc.nal.usda.gov/food-details/172181/nutrients (accessed on 30 April 2026)
172207	Cheese, cream, fat free	SR Legacy	Cream cheese (cheese)	fat free	baseline	Not selected	1	https://fdc.nal.usda.gov/food-details/172207/nutrients (accessed on 30 April 2026)
169079	Cheese, cream, low fat	SR Legacy	Cream cheese (cheese)	low fat	baseline	Not selected	16.67	https://fdc.nal.usda.gov/food-details/169079/nutrients (accessed on 30 April 2026)
2259796	Cheese, feta, whole milk, crumbled	Foundation	Feta (cheese)	whole milk	baseline	Not selected	19.08	https://fdc.nal.usda.gov/food-details/2259796/nutrients (accessed on 30 April 2026)
173439	Cheese, low fat, cheddar or colby	SR Legacy	Cheddar (cheese)	low fat	baseline	Not selected	7	https://fdc.nal.usda.gov/food-details/173439/nutrients (accessed on 30 April 2026)
172216	Cheese, Mexican, blend, reduced fat	SR Legacy	Mexican blend (cheese)	reduced fat	baseline	Not selected	19.4	https://fdc.nal.usda.gov/food-details/172216/nutrients (accessed on 30 April 2026)
168098	Cheese, monterey, low fat	SR Legacy	Monterey (cheese)	low fat	baseline	Not selected	21.6	https://fdc.nal.usda.gov/food-details/168098/nutrients (accessed on 30 April 2026)
171244	Cheese, mozzarella, low moisture, part-skim	SR Legacy	Low-moisture mozzarella	part-skim	baseline	Selected	19.78	https://fdc.nal.usda.gov/food-details/171244/nutrients (accessed on 30 April 2026)
170900	Cheese, mozzarella, low moisture, part-skim, shredded	SR Legacy	Low-moisture mozzarella	part-skim	baseline	Not selected	19.72	https://fdc.nal.usda.gov/food-details/170900/nutrients (accessed on 30 April 2026)
169051	Cheese, mozzarella, nonfat	SR Legacy	Mozzarella (other)	nonfat	baseline	Not selected	0	https://fdc.nal.usda.gov/food-details/169051/nutrients (accessed on 30 April 2026)
170847	Cheese, mozzarella, part skim milk	SR Legacy	Mozzarella (other)	part skim milk	baseline	Not selected	15.92	https://fdc.nal.usda.gov/food-details/170847/nutrients (accessed on 30 April 2026)
170845	Cheese, mozzarella, whole milk	SR Legacy	Mozzarella (other)	whole milk	baseline	Not selected	22.14	https://fdc.nal.usda.gov/food-details/170845/nutrients (accessed on 30 April 2026)
170846	Cheese, mozzarella, whole milk, low moisture	SR Legacy	Low-moisture mozzarella	whole milk	baseline	Selected	24.64	https://fdc.nal.usda.gov/food-details/170846/nutrients (accessed on 30 April 2026)
169050	Cheese, muenster, low fat	SR Legacy	Muenster (cheese)	low fat	baseline	Not selected	17.6	https://fdc.nal.usda.gov/food-details/169050/nutrients (accessed on 30 April 2026)
173452	Cheese, parmesan, dry grated, reduced fat	SR Legacy	Parmesan (cheese)	reduced fat	baseline	Not selected	20	https://fdc.nal.usda.gov/food-details/173452/nutrients (accessed on 30 April 2026)
172215	Cheese, provolone, reduced fat	SR Legacy	Provolone (cheese)	reduced fat	baseline	Not selected	17.6	https://fdc.nal.usda.gov/food-details/172215/nutrients (accessed on 30 April 2026)
171248	Cheese, ricotta, part skim milk	SR Legacy	Ricotta	part skim milk	baseline	Selected	7.91	https://fdc.nal.usda.gov/food-details/171248/nutrients (accessed on 30 April 2026)
746766	Cheese, ricotta, whole milk	Foundation	Ricotta	whole milk	baseline	Not selected	11	https://fdc.nal.usda.gov/food-details/746766/nutrients (accessed on 30 April 2026)
170851	Cheese, ricotta, whole milk	SR Legacy	Ricotta	whole milk	baseline	Selected: one USDA SR Legacy record from the chosen within-product illustrative series.	10.18	https://fdc.nal.usda.gov/food-details/170851/nutrients (accessed on 30 April 2026)
168141	Cheese, swiss, low fat	SR Legacy	Swiss (cheese)	low fat	baseline	Not selected	5.1	https://fdc.nal.usda.gov/food-details/168141/nutrients (accessed on 30 April 2026)
170897	Cheese, Swiss, nonfat or fat free	SR Legacy	Swiss (cheese)	nonfat or fat free	baseline	Not selected	0	https://fdc.nal.usda.gov/food-details/170897/nutrients (accessed on 30 April 2026)
2346384	Cottage cheese, full fat, large or small curd	Foundation	Cottage cheese (cheese)	full fat	baseline	Not selected	4.225	https://fdc.nal.usda.gov/food-details/2346384/nutrients (accessed on 30 April 2026)
2346385	Cream cheese, full fat, block	Foundation	Cream cheese (cheese)	full fat	baseline	Not selected	33.49	https://fdc.nal.usda.gov/food-details/2346385/nutrients (accessed on 30 April 2026)
171255	Cream, fluid, half and half	SR Legacy	Fluid cream	half and half	baseline	Not selected	11.5	https://fdc.nal.usda.gov/food-details/171255/nutrients (accessed on 30 April 2026)
170859	Cream, fluid, heavy whipping	SR Legacy	Fluid cream	heavy	baseline	Not selected	36.08	https://fdc.nal.usda.gov/food-details/170859/nutrients (accessed on 30 April 2026)
171308	Cream, half and half, lowfat	SR Legacy	Fluid cream	half and half, lowfat	baseline	Not selected	5	https://fdc.nal.usda.gov/food-details/171308/nutrients (accessed on 30 April 2026)
171256	Cream, sour, reduced fat, cultured	SR Legacy	Sour cream	reduced fat	baseline	Not selected	12	https://fdc.nal.usda.gov/food-details/171256/nutrients (accessed on 30 April 2026)
173444	Sour cream, fat free	SR Legacy	Sour cream	fat free	baseline	Not selected	0	https://fdc.nal.usda.gov/food-details/173444/nutrients (accessed on 30 April 2026)
173442	Sour cream, reduced fat	SR Legacy	Sour cream	reduced fat	baseline	Not selected	14.1	https://fdc.nal.usda.gov/food-details/173442/nutrients (accessed on 30 April 2026)
2346387	Cream, sour, full fat	Foundation	Sour cream	full fat	baseline	Not selected	17.99	https://fdc.nal.usda.gov/food-details/2346387/nutrients (accessed on 30 April 2026)
2259793	Yogurt, plain, whole milk	Foundation	Plain yogurt	whole milk	baseline	Not selected	4.484	https://fdc.nal.usda.gov/food-details/2259793/nutrients (accessed on 30 April 2026)
2259792	Buttermilk, low fat	Foundation	Buttermilk	low fat	baseline	Not selected	1.081	https://fdc.nal.usda.gov/food-details/2259792/nutrients (accessed on 30 April 2026)
170874	Milk, buttermilk, fluid, cultured, lowfat	SR Legacy	Buttermilk	low fat	baseline	Not selected	1.07	https://fdc.nal.usda.gov/food-details/170874/nutrients (accessed on 30 April 2026)
167697	Milk, buttermilk, fluid, cultured, reduced fat	SR Legacy	Buttermilk	reduced fat	baseline	Not selected	2	https://fdc.nal.usda.gov/food-details/167697/nutrients (accessed on 30 April 2026)
172225	Milk, buttermilk, fluid, whole	SR Legacy	Buttermilk	whole milk	baseline	Not selected	3.31	https://fdc.nal.usda.gov/food-details/172225/nutrients (accessed on 30 April 2026)
746772	Milk, lowfat, fluid, 1% milkfat, with added vitamin A and vitamin D	Foundation	Fluid milk	1% milkfat	baseline	Not selected	0.95	https://fdc.nal.usda.gov/food-details/746772/nutrients (accessed on 30 April 2026)
746778	Milk, reduced fat, fluid, 2% milkfat, with added vitamin A and vitamin D	Foundation	Fluid milk	2% milkfat	sepcific processed	Not selected	1.9	https://fdc.nal.usda.gov/food-details/746778/nutrients (accessed on 30 April 2026)
171265	Milk, whole, 3.25% milkfat, with added vitamin D	SR Legacy	Fluid milk	3.25% milkfat	sepcific processed	Not selected	3.25	https://fdc.nal.usda.gov/food-details/171265/nutrients (accessed on 30 April 2026)
746782	Milk, whole, 3.25% milkfat, with added vitamin D	Foundation	Fluid milk	3.25% milkfat	sepcific processed	Not selected	3.2	https://fdc.nal.usda.gov/food-details/746782/nutrients (accessed on 30 April 2026)
746776	Milk, nonfat, fluid, with added vitamin A and vitamin D (fat free or skim)	Foundation	Fluid milk	Skim/nonfat	baseline	Not selected	0.08	https://fdc.nal.usda.gov/food-details/746776/nutrients (accessed on 30 April 2026)
170873	Milk, lowfat, fluid, 1% milkfat, with added nonfat milk solids, vitamin A and vitamin D	SR Legacy	Fluid milk	1% milkfat	sepcific processed	Not selected	0.97	https://fdc.nal.usda.gov/food-details/170873/nutrients (accessed on 30 April 2026)
172193	Milk, reduced fat, fluid, 2% milkfat, with added nonfat milk solids, without added vitamin A	SR Legacy	Fluid milk	2% milkfat	sepcific processed	Not selected	1.98	https://fdc.nal.usda.gov/food-details/172193/nutrients (accessed on 30 April 2026)
170870	Milk, reduced fat, fluid, 2% milkfat, with added nonfat milk solids and vitamin A and vitamin D	SR Legacy	Fluid milk	2% milkfat	sepcific processed	Not selected	1.92	https://fdc.nal.usda.gov/food-details/170870/nutrients (accessed on 30 April 2026)
173441	Milk, fluid, 1% fat, without added vitamin A and vitamin D	SR Legacy	Fluid milk	1% fat	baseline	Selected	0.97	https://fdc.nal.usda.gov/food-details/173441/nutrients (accessed on 30 April 2026)
169868	Milk, fluid, nonfat, calcium fortified (fat free or skim)	SR Legacy	Fluid milk	nonfat	sepcific processed	Not selected	0.18	https://fdc.nal.usda.gov/food-details/169868/nutrients (accessed on 30 April 2026)
171268	Milk, lowfat, fluid, 1% milkfat, protein fortified, with added vitamin A and vitamin D	SR Legacy	Fluid milk	low fat/1% milkfat	sepcific processed	Not selected	1.17	https://fdc.nal.usda.gov/food-details/171268/nutrients (accessed on 30 April 2026)
170872	Milk, lowfat, fluid, 1% milkfat, with added vitamin A and vitamin D	SR Legacy	Fluid milk	low fat/1% milkfat	sepcific processed	Not selected	0.97	https://fdc.nal.usda.gov/food-details/170872/nutrients (accessed on 30 April 2026)
171271	Milk, nonfat, fluid, protein fortified, with added vitamin A and vitamin D (fat free and skim)	SR Legacy	Fluid milk	nonfat	sepcific processed	Not selected	0.25	https://fdc.nal.usda.gov/food-details/171271/nutrients (accessed on 30 April 2026)
171269	Milk, nonfat, fluid, with added vitamin A and vitamin D (fat free or skim)	SR Legacy	Fluid milk	nonfat	sepcific processed	Not selected	0.08	https://fdc.nal.usda.gov/food-details/171269/nutrients (accessed on 30 April 2026)
173432	Milk, nonfat, fluid, without added vitamin A and vitamin D (fat free or skim)	SR Legacy	Fluid milk	nonfat	baseline	Selected	0.18	https://fdc.nal.usda.gov/food-details/173432/nutrients (accessed on 30 April 2026)
170871	Milk, reduced fat, fluid, 2% milkfat, protein fortified, with added vitamin A and vitamin D	SR Legacy	Fluid milk	reduced fat/2% milkfat	sepcific processed	Not selected	1.98	https://fdc.nal.usda.gov/food-details/170871/nutrients (accessed on 30 April 2026)
171267	Milk, reduced fat, fluid, 2% milkfat, with added vitamin A and vitamin D	SR Legacy	Fluid milk	reduced fat/2% milkfat	sepcific processed	Not selected	1.98	https://fdc.nal.usda.gov/food-details/171267/nutrients (accessed on 30 April 2026)
172205	Milk, reduced fat, fluid, 2% milkfat, without added vitamin A and vitamin D	SR Legacy	Fluid milk	reduced fat/2% milkfat	baseline	Selected	1.98	https://fdc.nal.usda.gov/food-details/172205/nutrients (accessed on 30 April 2026)
171270	Milk, nonfat, fluid, with added nonfat milk solids, vitamin A and vitamin D (fat free or skim)	SR Legacy	Fluid milk	Skim/nonfat	sepcific processed	Not selected	0.25	https://fdc.nal.usda.gov/food-details/171270/nutrients (accessed on 30 April 2026)
172217	Milk, whole, 3.25% milkfat, without added vitamin A and vitamin D	SR Legacy	Fluid milk	whole/3.25% milkfat	baseline	Selected	3.27	https://fdc.nal.usda.gov/food-details/172217/nutrients (accessed on 30 April 2026)
170903	Yogurt, Greek, plain, lowfat	SR Legacy	Greek yogurt	lowfat	baseline	Selected	1.92	https://fdc.nal.usda.gov/food-details/170903/nutrients (accessed on 30 April 2026)
330137	Yogurt, Greek, plain, nonfat	Foundation	Greek yogurt	nonfat	baseline	Not selected	0.37	https://fdc.nal.usda.gov/food-details/330137/nutrients (accessed on 30 April 2026)
170894	Yogurt, Greek, plain, nonfat (Includes foods for USDA’s Food Distribution Program)	SR Legacy	Greek yogurt	nonfat	baseline	Selected	0.39	https://fdc.nal.usda.gov/food-details/170894/nutrients (accessed on 30 April 2026)
171304	Yogurt, Greek, plain, whole milk	SR Legacy	Greek yogurt	whole milk	baseline	Selected	5	https://fdc.nal.usda.gov/food-details/171304/nutrients (accessed on 30 April 2026)
2259794	Yogurt, Greek, plain, whole milk	Foundation	Greek yogurt	whole milk	baseline	Not selected	4.394	https://fdc.nal.usda.gov/food-details/2259794/nutrients (accessed on 30 April 2026)
170886	Yogurt, plain, low fat	SR Legacy	Plain yogurt	lowfat	baseline	Selected	1.55	https://fdc.nal.usda.gov/food-details/170886/nutrients (accessed on 30 April 2026)
2647437	Yogurt, plain, nonfat	Foundation	Plain yogurt	nonfat	baseline	Not selected	0.0875	https://fdc.nal.usda.gov/food-details/2647437/nutrients (accessed on 30 April 2026)
170887	Yogurt, plain, skim milk	SR Legacy	Plain yogurt	skim	baseline	Selected	0.18	https://fdc.nal.usda.gov/food-details/170887/nutrients (accessed on 30 April 2026)
171284	Yogurt, plain, whole milk	SR Legacy	Plain yogurt	whole milk	baseline	Selected	3.25	https://fdc.nal.usda.gov/food-details/171284/nutrients (accessed on 30 April 2026)

Note: Each row represents one USDA food record, not a mean of multiple products. SFA, saturated fatty acids; dry matter = 100 − water (g/100 g). Fluid-milk records are specified as without added vitamins A and D. Milk-fat descriptors follow USDA product names. Values are illustrative food composition data, not results from matched experimental products. Source: USDA FoodData Central, SR Legacy (April 2018 release); FDC IDs are shown for traceability. The presentation rationale indicates whether each USDA record was selected for inclusion in Table 2 and Table 3.
